# Evaluation of Deep Eutectic Solvents for Cryopreservation of the Fish Pathogen *Saprolegnia parasitica*

**DOI:** 10.3390/mps9030085

**Published:** 2026-06-01

**Authors:** Sara Delimar, Ela Šarić, Marina Cvjetko Bubalo, Ana Bielen

**Affiliations:** Department of Biochemical Engineering, University of Zagreb Faculty of Food Technology and Biotechnology, 10000 Zagreb, Croatia; sara.delimar@pbf.unizg.hr (S.D.); ela.saric@pbf.unizg.hr (E.Š.)

**Keywords:** aquaculture disease, cryoprotectant screening, deep eutectic solvents, freezing protocol optimization, oomycetes, post-thaw recovery, *Saprolegnia*

## Abstract

*Saprolegnia parasitica* (Oomycota) causes saprolegniosis and poses significant ecological and economic challenges in aquaculture. Experimental research on this pathogen is constrained by the lack of reliable long-term preservation methods, as routine maintenance by serial subculturing is labor-intensive and may result in genetic and phenotypic instability. Deep eutectic solvents (DESs), tunable low-melting mixtures, have recently gained attention as alternative cryoprotectants. However, their application has not been evaluated in oomycetes. Here, twelve glycerol-based two- and multicomponent DESs were assessed for cryopreservation of *S. parasitica* at −80 °C and compared with glycerol as a conventional cryoprotectant. Cryopreservation efficiency was assessed based on post-thaw survival and mycelial regeneration. Several two-component DESs, particularly glycerol-trehalose, supported 100% survival and high post-thaw mycelial regeneration, performing comparably to glycerol under the tested conditions. Shorter pre-incubation (30 min vs. 1 h and 3 h) and controlled-rate freezing (vs. direct freezing) significantly improved post-thaw growth. Although survival remained 100% under optimized conditions, extending storage from 7 to 32 days significantly reduced mycelial regeneration in the glycerol–trehalose treatment, indicating that survival alone, as done in existing literature, does not reflect physiological recovery. Overall, our results support the use of selected DESs as alternative cryoprotectants in oomycetes and contribute to the development of cryopreservation strategies for *S. parasitica*.

## 1. Introduction

Water moulds of the genus *Saprolegnia* cause saprolegniosis, a disease responsible for significant economic and ecological losses in aquatic ecosystems. Saprolegniosis can infect all developmental stages of salmonids and other fish, from eggs and larvae to juveniles and adults, often with high mortality rates. Among *Saprolegnia* spp., *Saprolegnia parasitica* is considered one of the most destructive species [1,2]. Saprolegniosis is a serious problem in salmonid aquaculture, where insufficient disease control measures and poor breeding conditions frequently lead to mass infections of fish eggs [1,3]. Consequently, outbreaks of saprolegniosis result in significant economic losses worldwide, with average annual losses exceeding 10% and, in severe outbreaks, reaching up to 50% [1,4,5].

The experimental work on *Saprolegnia* spp., necessary for the reproducible pathogenesis studies and development of improved management strategies, is dependent on the long-term laboratory preservation of genetically stable isolates. Oomycetes are commonly maintained short-term through serial subculturing on solid media [6,7,8], although repeated passaging is labor-intensive, prone to contamination, and often leads to genotypic and phenotypic alterations, as demonstrated for *S. parasitica* and other microbial taxa [8,9,10]. However, standardized protocols for preservation of oomycetes for longer periods remain insufficiently developed [11]. Alternative oomycete storage methods, such as maintenance in sterile water or under mineral oil, provide medium-term preservation (commonly 1–7 years depending on the species) but show variable survival across species and may be associated with reduced growth performance after recovery [6,12,13,14,15]. Cryopreservation at −80 °C, in liquid nitrogen or vapour-phase nitrogen storage, represents the most promising long-term strain preservation strategy, as it requires minimal maintenance, halts metabolic activity (i.e., minimizes evolutionary drift), and reduces contamination risk [16,17].

One of the prerequisites for successful cryopreservation is the mitigation of ice crystal-induced cellular damage. This is usually achieved by adding cryoprotectants that help reduce ice formation in cell suspension [16]. To date, dimethyl sulfoxide (DMSO) and glycerol have been the most commonly tested cryoprotectants for oomycetes and other organisms [6,11,16,18,19,20], but both show cytotoxicity at the concentrations used for cryopreservation, and DMSO is additionally associated with genotoxicity [21]. These limitations underscore the need to identify and evaluate alternative cryoprotective agents for oomycetes.

Deep eutectic solvents (DESs) have recently emerged as promising alternative cryoprotectants [22,23,24]. DESs are mixtures of two or more components that form a eutectic system, characterized by a significant melting point depression compared to the individual constituents, typically resulting in a liquid state at ambient temperatures [25]. Their preparation typically involves simple mixing of components under mild heating, and their physicochemical properties can be tuned by varying their composition. Depending on the constituents used, certain DES formulations have been reported to exhibit lower cytotoxicity and improved environmental profiles compared to conventional organic solvents [22,26,27]. Notably, DESs can be formed by mixing naturally occurring primary metabolites, such as trehalose, glucose, sorbitol and proline, which have been associated with tolerance of some animals for very low temperatures [28].

Despite the relevance of cryopreservation for oomycete research, three key gaps remain. First, standardized cryopreservation protocols for oomycetes, including *Saprolegnia* spp., are still insufficiently developed, as also emphasized in recent reviews of fungal and oomycete cryopreservation [29]. Existing protocols mainly rely on conventional cryoprotectants such as glycerol and DMSO, storage at −80 °C or in liquid nitrogen, and direct or controlled-rate freezing, but reported post-freezing survival rates remain inconsistent and often depend on strain, cryoprotectant composition, freezing protocol, and storage duration [6,11,16,18,19,20]. Second, while these conventional approaches provide the current reference framework, DES-based cryoprotectants have not been evaluated in oomycetes. Third, most studies assess post-thaw survival only [6,16,18,19,20], while the potential effects of freezing on mycelial regeneration and growth dynamics were rarely examined. These gaps highlight the need for improved and systematically evaluated cryopreservation protocols for oomycetes, including target species like *S. parasitica*.

It was recently demonstrated that DESs comprising biologically relevant osmolytes, such as sugars (e.g., glucose, trehalose and sucrose), polyols (glycerol and sorbitol) and betaine can act as effective cryoprotective agents in the vitrification of mammalian cells [22,23,24,26]. This raised the question of whether osmolyte-based DES formulations, originally developed or characterized in non-oomycete systems, could also support cryopreservation of the oomycete *S. parasitica* after freezing at −80 °C. We hypothesized that selected osmolyte-based DES formulations would provide cryoprotection comparable to glycerol, a reference cryoprotectant commonly used in oomycete cryopreservation protocols [11,30], as reflected in post-thaw survival and mycelial regeneration. Accordingly, this study aimed to systematically screen selected osmolyte-based DES formulations for *S. parasitica* cryopreservation and to evaluate key protocol parameters affecting post-thaw recovery. To our knowledge, this is the first study to evaluate DES-based cryoprotectants for oomycete cryopreservation, with the novelty lying in their biological application rather than in the development of new DES chemistry.

## 2. Materials and Methods

### 2.1. Chemicals

Betaine was purchased from Thermo Fisher Scientific (Geel, Belgium). D-fructose was sourced from Lachner (Neratovice, Czech Republic). Dimethylsulfoniopropionate, L-α-glycerophosphorylcholine and sorbitol were obtained from Biosynth (Bratislava, Slovakia). Ectoine, isopropanol, L-proline, oxolinic acid, penicillin G, sucrose, sarcosine, taurine, trehalose, and urea were purchased from Sigma-Aldrich (St. Louis, MO, USA). Glycerol was obtained from Merck (Darmstadt, Germany). All chemicals were used without further purification.

### 2.2. Preparation of Deep Eutectic Solvents

Deep eutectic solvents (DESs) that were prepared and tested in this study, as well as their constituent components, are listed in Table 1. The molar ratios for each DES formulation were selected based on established literature protocols [28].

DESs were prepared by combining the individual components in specific molar ratios (Table 1), following standard protocols [31]. The required amounts of each constituent were quantitatively weighed into Falcon tubes. When applicable, a precise volume of distilled water was added to the mixture to facilitate eutectic formation (Table 1). The tubes were then placed in an incubator shaker and stirred at a controlled temperature up to 60 °C for up to 3 h, or until a clear, homogeneous liquid without visible crystals was obtained. To ensure stability and consistency across experiments, the DESs were stored at room temperature. Prior usage, DESs were diluted with sterile distilled water to a final volume fraction of 10% (*v*/*v*). In addition to the DESs, a 10% (*v*/*v*) glycerol solution was prepared by mixing the appropriate ratio of glycerol and sterile distilled water.

### 2.3. Culture Conditions

In this study, an isolate of the water mold *Saprolegnia parasitica* Coker CBS 223.65, originally isolated from pike (*Esox lucius*), was used (provided by R. Galuppi, University of Bologna, Bologna, Italy). *Saprolegnia parasitica* isolate was cultivated at 14 °C on solid GY medium [32], supplemented with penicillin G (6 mg/L) and oxolinic acid (10 mg/L) to suppress the bacterial growth [33]. Inoculation of *S. parasitica* cultures was performed by cutting a piece of mycelium-overgrown agar from the edge of an actively growing colony and placing it in the middle of the fresh GY plate. Such cultures were routinely maintained by serial passaging every 10–15 days and stored at 4 °C.

### 2.4. Determination of Optimal Cryopreservation Conditions

This study consisted of four sequential experiments in which individual cryopreservation parameters were systematically varied to optimize cryopreservation conditions for *S. parasitica* (Table 2). Agar plugs (5 × 5 mm) excised from the edge of an actively growing mycelium were transferred into 2 mL cryotubes containing 1.5 mL of either 10% glycerol or a selected 10% DES under sterile conditions. The samples were pre-incubated at 14 °C and subsequently frozen at −80 °C. After the designated storage period, samples were thawed in a water bath (JB Nova, Grant Instruments, Shepreth, UK) at 22 °C until completely thawed. Agar plugs overgrown with *S. parasitica* mycelium were then rinsed with sterile distilled water to remove residual solvent, transferred onto solid GY medium, and incubated at 14 °C for 7 days. Survival was determined based on the number of replicates that showed visible mycelial growth after thawing. Experiment 1 included three biological replicates per treatment, whereas Experiments 2–4 included five biological replicates per treatment. Post-thaw mycelial regeneration was quantified by measuring the mycelial radius after incubation. Petri dishes were scanned together with millimetre paper and analysed using ImageJ software v1.53. The image scale was calibrated using the millimetre paper by setting 1 cm as the known distance. For each biological replicate, the mycelial radius was measured along a line crossing the agar plug and the colony. Two measurements were taken from the edge of the agar plug to the colony margin in opposite directions, and these two measurements were treated as technical measurements. Their mean value was used for statistical analysis.

Based on the results of Experiment 1, Gly and Gly:Treh were used as cryoprotectants in subsequent experiments. In Experiment 2, freezing methods were compared, including direct freezing at −80 °C and controlled-rate freezing using a Mr. Frosty™ container (Nalgene, Rochester, NY, USA) filled with isopropanol. For controlled-rate freezing, samples were placed at −80 °C for approximately 40 min to achieve gradual cooling at an approximate rate of −1 °C/min, after which they were removed from the container and stored at −80 °C. Since controlled-rate freezing resulted in significantly higher post-thaw mycelial growth, this freezing method was used in Experiments 3 and 4. In Experiment 3, pre-incubation duration was varied and based on the results obtained, the selected pre-incubation duration of 30 min was applied in the final experiment. In Experiment 4, the effect of storage duration (7 vs. 32 days) on post-thaw mycelial growth was assessed.

### 2.5. Statistical Analyses

Statistical analyses were performed in Python 3.12.13 using Google Colaboratory, with pandas 2.2.2, NumPy 2.0.2, SciPy 1.16.3, scikit-posthocs 0.13.0, and openpyxl 3.1.5. Biological replicate values were used as the unit of analysis. For each biological replicate, two opposite-direction radius measurements (technical replicates) were first averaged to obtain one mycelial-radius value per biological replicate. Replicates showing no visible post-thaw mycelial regrowth were not treated as missing data, but were assigned a mycelial radius of 0 cm, because the absence of regrowth represented a valid biological outcome of cryopreservation failure. These zero values were therefore included in group summaries and statistical analyses of post-thaw mycelial regeneration. Because of the small sample size, non-normal data structure, and the presence of zero-inflated datasets in some treatments, differences among treatment groups were assessed using the non-parametric Kruskal–Wallis test. When the overall test was significant, post hoc pairwise comparisons were performed using Dunn’s test with Holm correction. Statistical significance was set at *p* < 0.05.

## 3. Results

### 3.1. Experiment 1—Selection of the Optimal DES Compared to Glycerol

**Figure 1:**
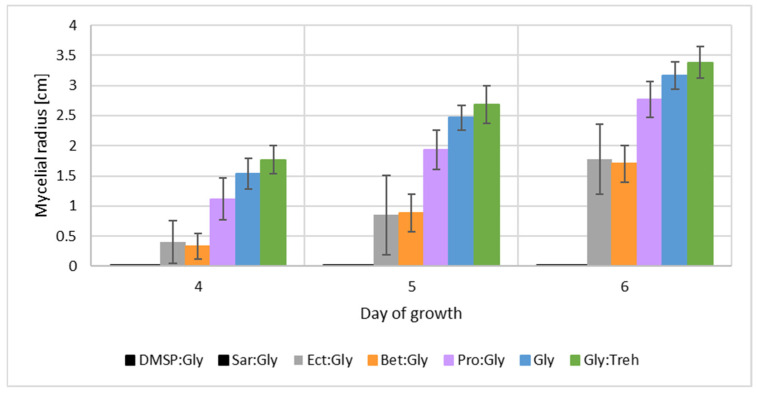
Mycelial growth of *S. parasitica* after seven days of freezing at −80 °C using 10% (*v*/*v*) glycerol and two-component DESs. Results are presented as mean ± standard deviation (*n* = 3). Large standard deviations reflect either differences in post-thaw mycelial regeneration among biological replicates with complete visible survival (3/3) or, in treatments with incomplete survival (1/3 or 2/3), zero-radius values in replicates without visible post-thaw regrowth.

**Figure 2:**
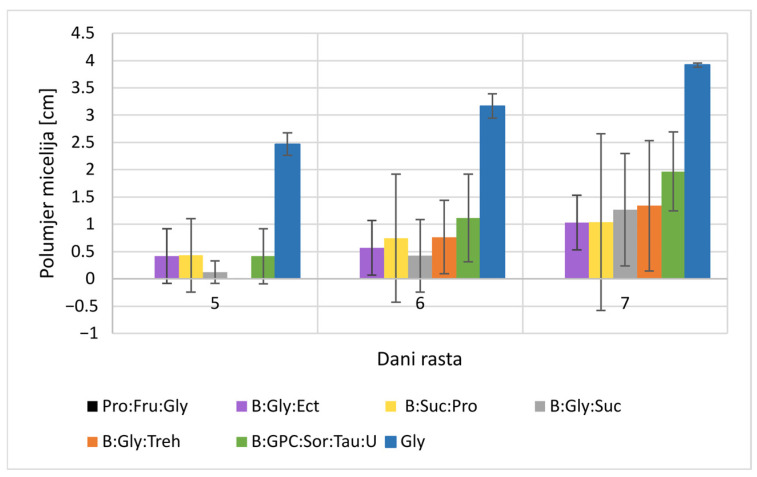
Mycelial growth of *S. parasitica* after seven days of freezing at −80 °C using 10% (*v*/*v*) glycerol and multicomponent DESs. Results are presented as mean ± standard deviation (*n* = 3). Large standard deviations reflect either differences in post-thaw mycelial regeneration among biological replicates with complete visible survival (3/3) or, in treatments with incomplete survival (1/3 or 2/3), zero-radius values in replicates without visible post-thaw regrowth.

The cryoprotective efficiency of various DESs in comparison with glycerol for the cryopreservation of *S. parasitica* was analysed. Differences in *S. parasitica* post-thaw survival (Table 3) and mycelial regeneration (Figure 1 and Figure 2; Datasets S1 and S2) were observed among the tested cryoprotectants. Statistically significant effect of different applied solvents was detected on day 6 of growth (H = 29.52, *p* = 0.0033). However, Dunn’s post hoc test with Holm correction did not identify significant pairwise differences between individual cryoprotectants (all adjusted *p* ≥ 0.0997).

Descriptively, the best cryoprotective activity was observed for glycerol, most two-component DESs (Pro:Gly, Ect:Gly, B:Gly, Gly:Treh), and one multicomponent DES (B:GPC:Sor:Tau:U), as survival was observed in all three replicates and post-thaw mycelial radius values were highest. In comparison, treatment with the remaining solvents resulted in reduced or no survival. The highest cryoprotective performance was measured for Gly:Treh (mean mycelial radius at day 6 post-thaw 3.383 ± 0.256 cm), followed by Gly (3.167 ± 0.225 cm), and Pro:Gly (2.767 ± 0.301 cm) (Figure 1; Datasets S1 and S2). No statistically significant difference in mycelial growth was detected among these three cryoprotectants (*p* > 0.05). Given the consistently high survival and growth observed with Gly:Treh and Gly, these two solvents were selected for further optimization experiments.

### 3.2. Experiment 2—Effect of Freezing Method

**Figure 3:**
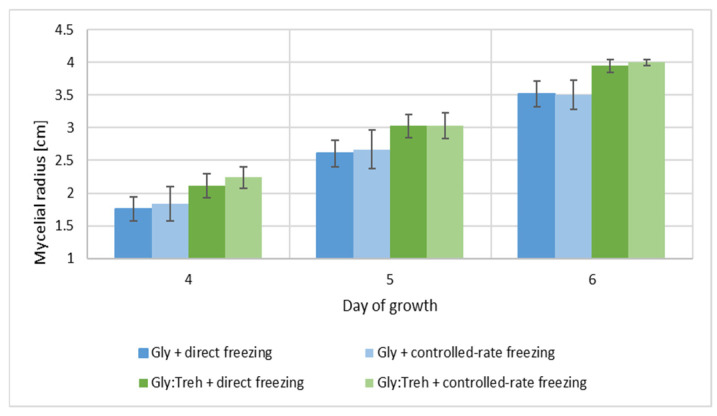
Mycelial growth of *S. parasitica* after seven days of freezing at −80 °C using 10% (*v*/*v*) glycerol (Gly) and glycerol:trehalose (Gly:Treh), following direct or controlled-rate freezing. Results are presented as mean ± standard deviation (*n* = 5).

The Kruskal–Wallis test revealed significant differences in mycelial growth among cryoprotectant–freezing treatment combinations on day 6 (H = 15.58, *p* = 0.0014). Dunn’s post hoc test with Holm correction showed no significant differences between Gly:Treh and glycerol within the same freezing method, although Gly:Treh-treated samples showed larger mycelial radii on all measurement days. However, controlled-rate freezing resulted in significantly higher mycelial growth than direct freezing for both Gly:Treh (*p* = 0.0130) and glycerol (*p* = 0.0476) (Figure 3; Datasets S1 and S2). Thus, under the conditions tested, the freezing method had a stronger statistically supported effect on post-thaw mycelial regeneration than the difference between Gly:Treh and glycerol. Therefore, controlled-rate freezing was used in subsequent experiments.

### 3.3. Experiment 3—Effect of Pre-Incubation Duration

**Figure 4:**
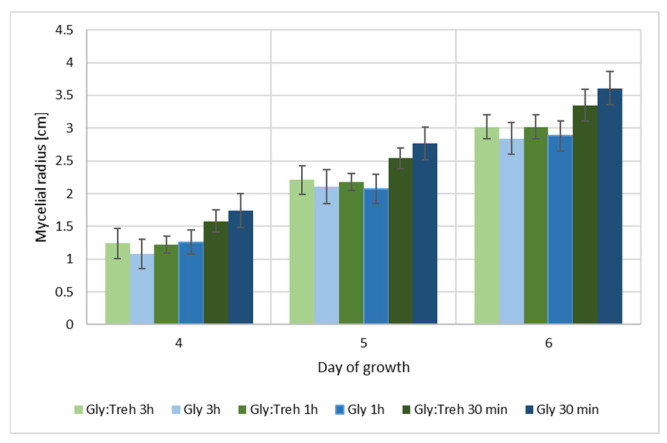
Mycelial growth of *S. parasitica* after seven days of freezing at −80 °C using 10% (*v*/*v*) glycerol (Gly) and glycerol:trehalose (Gly:Treh) with different pre-incubation durations at 14 °C (30 min, 1 h, and 3 h). Results are presented as mean ± standard deviation (*n* = 5).

The Kruskal–Wallis test revealed significant differences in mycelial growth among cryoprotectant–pre-incubation treatment groups on day 6 (H = 17.91, *p* = 0.0031). Dunn’s post hoc test with Holm correction showed that, for glycerol, 30 min pre-incubation resulted in significantly higher mycelial growth than 1 h (*p* = 0.0214) or 3 h (*p* = 0.0130) pre-incubation (Figure 4; Datasets S1 and S2). In contrast, no significant differences among pre-incubation durations were detected for Gly:Treh (all adjusted *p* ≥ 0.8457). In the 30 min pre-incubation treatment, post-thaw mycelial growth was descriptively higher with glycerol, whereas after 1 h and 3 h pre-incubation, higher mean values were observed with Gly:Treh; however, none of these cryoprotectant-specific differences were statistically significant (all adjusted *p* ≥ 0.1554). Altogether, based on the obtained results, 30 min pre-incubation was selected for the final experiment, as it produced the highest growth with both cryoprotectants.

### 3.4. Experiment 4—Effect of Storage Duration

**Figure 5:**
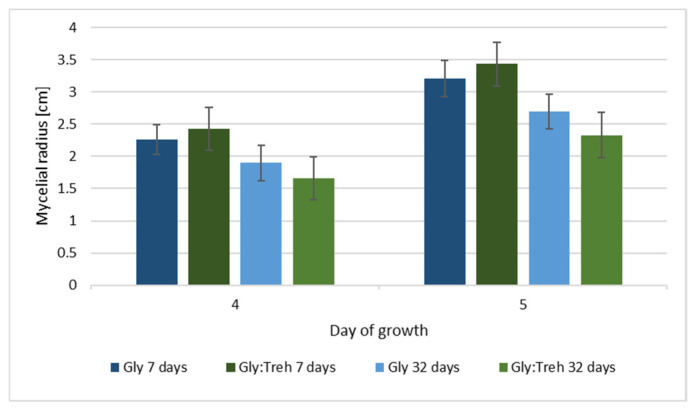
Mycelial growth of *S. parasitica* after storage at −80 °C for 7 and 32 days using 10% (*v*/*v*) glycerol (Gly) and glycerol:trehalose (Gly:Treh). Results are presented as mean ± standard deviation (*n* = 5).

Survival of *S. parasitica* was 100% in all tested conditions, but a statistically significant overall effect of storage duration and cryoprotectant used on the post-thaw mycelial growth was detected (H = 14.62, *p* = 0.0022, compared using post-thaw mycelium radius measurements after 5 days). Dunn’s post hoc test with Holm correction showed that storage for 32 days significantly reduced post-thaw mycelial growth compared with 7 days only for Gly:Treh (*p* = 0.0030; Figure 5; Datasets S1 and S2). No significant difference was detected between 7 and 32 days of storage for glycerol (*p* = 0.3612), nor between glycerol and Gly:Treh within the same storage duration (*p* ≥ 0.4775). Descriptively, Gly:Treh showed higher post-thaw mycelial regeneration after 7 days, whereas glycerol showed higher regeneration after 32 days.

## 4. Discussion

To our knowledge, this is the first study evaluating the effect of DESs on the cryopreservation performance of oomycetes. Among the twelve tested DESs, the glycerol:trehalose formulation supported 100% *S. parasitica* survival, followed by the highest post-thaw mycelial regeneration rate, performing comparably to glycerol as a conventional cryoprotectant. Controlled-rate freezing improved cryopreservation outcomes compared to direct freezing, whereas shorter pre-incubation (30 min vs. 1 h and 3 h) promoted faster post-thaw growth, particularly with glycerol. However, extending short-term storage from 7 to 32 days resulted in slower mycelial regeneration, underscoring the need for further optimization of *S. parasitica* cryopreservation protocols.

Among the tested cryoprotectants, glycerol:trehalose (Gly:Treh) and glycerol alone showed the most consistent cryoprotective performance for *S. parasitica*, while pro-line:glycerol (Pro:Gly) emerged as the most effective alternative DES. Importantly, the relative performance of Gly:Treh and glycerol was condition-dependent: Gly:Treh showed descriptively higher mean mycelial radius in Experiments 1–3, whereas glycerol showed descriptively higher regeneration after 32 days of storage in Experiment 4; however, no statistically significant differences between Gly:Treh and glycerol were detected under the same treatment conditions.

Previous studies have demonstrated the cryoprotective potential of proline:glycerol, trehalose:glycerol, proline:glucose, and proline:sorbitol DESs for various cell types, including mammalian cell lines and the yeast *Saccharomyces cerevisiae* [21,24,27,34,35,36,37,38]. The observed performance of Gly:Treh and Pro:Gly may be interpreted in the context of established cryoprotective mechanisms of their constituents. Trehalose is widely recognized as a cryoprotective osmolyte that contributes to protein and membrane stabilization during cryopreservation-related stress [39], while proline has been reported to improve post-thaw recovery in other biological systems, likely through osmoprotective and intracellular stress-protective effects [40]. Glycerol, which was present in the most successful DES formulations and was used here as the reference cryoprotectant, is a conventional cryoprotectant in oomycete preservation protocols [6,11,16,18,19,20]. Thus, the observed DES performance may reflect the combined contribution of their individual constituents and mixture properties. This is consistent with the broader concept that naturally occurring osmolytes often function in mixtures rather than in isolation, and that such mixtures may contribute to stress protection through combined physicochemical and biological effects [28]. However, the present study did not directly test the underlying mechanisms or potential synergistic effects, which should be addressed in future work.

In contrast, several DESs evaluated in this study were tested for their cryoprotective effect for the first time. Among the multicomponent DESs, the most promising results were obtained with B:GPC:Sor:Tau:U, a DES whose composition corresponds to the osmolyte profile found in the rabbit kidney [28]. This is consistent with the concept that naturally occurring osmolyte mixtures may form DES-like systems with stress-protective properties [31]. However, because B:GPC:Sor:Tau:U was assessed only in the initial screening experiment, its apparent cryoprotective potential should be validated in further experiments.

Physicochemical properties, such as pH, may also contribute to DES cryoprotective performance. Except for DMSP:Gly, the pH values of the tested DES formulations ranged from 6 to 8, with no clear relationship between pH and cryoprotective activity within this range. However, the highly acidic DMSP:Gly formulation (pH 0.95 ± 0.18) was detrimental to *S. parasitica* survival, indicating that extreme acidity can strongly limit cryoprotective performance. This is consistent with the known sensitivity of *S. parasitica* to pH values below 4 [41,42].

While cryoprotectant composition and properties clearly influenced post-thaw performance, procedural parameters related to the freezing regime (i.e., direct vs. controlled freezing) may also affect cryopreservation outcomes [16,18,19,20,27]. In the present study, controlled-rate freezing slightly but significantly increased post-thaw mycelial growth compared to direct freezing. In oomycete literature, controlled-rate freezing has frequently been reported to improve survival, presumably by reducing intracellular ice formation; however, its beneficial effect is not universal and appears to be strain- and protocol-dependent, with variable outcomes reported across studies [16,20]. For instance, Nishii and Nakagiri [16] reported 100% survival of *S. parasitica*, *Phytophthora* spp. and *Pythium* spp. when controlled-rate freezing was employed. In contrast, MacAulay [20] observed reduced survival of *S. parasitica* under controlled-rate freezing conditions, while achieving 100% survival using direct freezing. This highlights the need for species- and strain-specific development of cryopreservation protocols in oomycetes.

The duration of pre-incubation in the presence of a cryoprotectant represents another critical parameter influencing post-thaw recovery. Pre-incubation is generally required to allow sufficient penetration of the cryoprotectant into cells; however, prolonged exposure may enhance cytotoxic effects. Therefore, the optimal duration of pre-incubation depends on the type of cells and the concentration and type of cryoprotectant used [37,38,43]. In the present study, shorter pre-incubation (30 min) resulted in the highest mean post-thaw mycelial regeneration for both glycerol and Gly:Treh. However, this effect was statistically significant only for glycerol. These results suggest that prolonged exposure to glycerol may negatively affect post-thaw recovery of *S. parasitica*, potentially reflecting cytotoxic effects associated with longer cryoprotectant exposure. The weaker decline observed for Gly:Treh is consistent with the possibility that this DES formulation may be less detrimental during longer pre-incubation, but this interpretation remains indirect because cytotoxicity was not directly measured. In line with our findings, studies on mammalian cells have shown that optimal pre-incubation durations ranged from 30 min to 1 h, depending on the DES used, while further extension resulted in reduced post-thaw survival [37,38]. Previous findings also reported reduced cytotoxicity of Gly:Treh compared to glycerol or trehalose alone in L929 mouse fibroblasts, attributed to a synergistic protective effect within the DES [34]. Whether such lower cytotoxicity or synergistic protection similarly applies to *S. parasitica* requires direct testing.

Beyond parameters acting prior to freezing, the duration of cryogenic storage itself is an important parameter that influences post-thaw survival and recovery [11,44,45]. In this study, in addition to survival, which remained 100% in all experiments where Gly and Gly:Treh were used, the effect of the time of storage at −80 °C on the post-thaw mycelial regeneration of *S. parasitica* was also investigated. The results showed reduced post-thaw mycelial growth after 32 days of storage compared to 7 days of storage, although this reduction was statistically significant only for Gly:Treh. These results indicate that post-thaw recovery may already be affected over the short storage interval tested, despite the preserved 100% visible post-thaw regrowth, in a cryoprotectant-dependent manner. The relatively high variability observed in some treatments during the initial DES screening also supports this interpretation. For example, in the Ect:Gly treatment, all three biological replicates showed visible post-thaw regrowth, but post-thaw mycelial regeneration varied substantially among replicates. This indicates that visible survival does not necessarily correspond to uniform post-thaw regeneration, reinforcing the need to assess recovery quality in addition to binary survival. However, meaningful comparison with previous studies is not possible, as available literature on oomycete cryopreservation reports only survival and does not quantify post-thaw regeneration dynamics [11,16,20]. For example, Nishii and Nakagiri [16] reported 100% survival of *S. parasitica* after six months of cryopreservation in 10% glycerol or 10% DMSO, while MacAulay [20] also observed complete survival after 24 days using the same concentrations. In contrast, extended storage for one year in 10% glycerol resulted in reduced survival (66%) of *S. parasitica* [11].

## 5. Conclusions

In conclusion, this study provides the first systematic evaluation of DESs for the development of *S. parasitica* cryopreservation protocols. As an initial study with a defined experimental scope, it focused on selected glycerol-based DES formulations, glycerol as a reference cryoprotectant, −80 °C storage, and key protocol parameters including freezing method and pre-incubation duration over short-term storage intervals of 7 and 32 days. The results demonstrate the potential of selected DES formulations as cryoprotectants for aquatic oomycetes, while also highlighting the need for further optimization of protocols to preserve not only post-thaw survival but also regenerative capacity of *S. parasitica* during storage beyond the short-term intervals tested here.

Future work should expand this scope by including additional controls, such as DMSO and freezing without cryoprotectant, testing a wider range of DES formulations and concentrations, validating protocols over longer storage periods, comparing −80 °C with lower-temperature storage approaches (such as liquid nitrogen or vapour-phase nitrogen) and assessing applicability across additional oomycete genera, species and strains. Although visible post-thaw mycelial growth, as used here, is a practical indicator of cryopreservation outcome, it does not capture all forms of sublethal cellular damage or physiological impairment. Therefore, future work should complement this approach with additional viability or physiological assays.

Finally, building on the concept that naturally occurring multi-osmolyte cocktails can form deep eutectic systems [28], future research could adopt a bioinspired strategy for the rational design of cryoprotective DESs tailored to oomycetes. This could be achieved by combining oomycete genome mining to assess the genomic potential for osmolyte biosynthesis and transport with targeted metabolomic analyses (e.g., LC–MS-based profiling) to identify osmolytes accumulating under low-temperature stress. Bioinspired DES formulations based on this approach should reflect the intrinsic stress physiology of oomycetes, for which consistent long-term cryopreservation remains technically challenging [11,20]. In the wider context, improved DES-based cryopreservation protocols could support more reliable maintenance of aquatic oomycete culture collections, thereby facilitating reproducible research on oomycete pathogens and contributing indirectly to aquaculture disease management.

## Figures and Tables

**Table 1:** Composition, molar ratios, and water content of the deep eutectic solvents (DESs) used in this study.

Deep Eutectic Solvent	Abbreviation	Component Molar Ratio	Water Content [%]	pH
Two-component DESs:				
proline:glycerol	Pro:Gly	1:3	/	6.02 ± 0.09
ectoin:glycerol	Ect:Gly	1:2	20	6.52 ± 0.02
betaine:glycerol	B:Gly	1:2	10	7.76 ± 0.06
glycerol:trehalose	Gly:Treh	30:1	/	6.12 ± 0.14
sarcosine:glycerol	Sar:Gly	1:2	20	6.45 ± 0.04
dimethylsulfoniopropionate:glycerol	DMSP:Gly	1:2	20	0.95 ± 0.18
Multicomponent DESs:				
betaine:glycerol:sucrose	B:Gly:Suc	2:3:1	10	7.09 ± 0.05
betaine:glycerol:ectoin	B:Gly:Ect	1:3:2	/	7.12 ± 0.09
betaine:sucrose:proline	B:Suc:Pro	5:2:2	20	7.45 ± 0.11
proline:fructose:glycerol	Pro:Fru:Gly	1:1:1	12	6.88 ± 0.15
betaine:glycerol:trehalose	B:Gly:Treh	2:3:1	13	8.0 ± 0.08
betaine:glycerylphosphorylcholine:sorbitol:taurine:urea	B:GPC:Sor:Tau:U	1:2.8:3.1:0.1:7.1	7	7.55 ± 0.02

**Table 2:** Overview of experimental conditions tested for optimization of *S. parasitica* cryopreservation.

Experiment	Solvent (10% *v*/*v*)	Duration of Pre-Incubation at 14 °C	Freezing Method	Storage Duration at −80 °C
1. Selection of optimal DES compared to glycerol	Gly Pro:Gly Ect:Gly B:Gly Gly:Treh Sar:Gly DMSP:Gly B:Gly:Suc B:Gly:Ect B:Suc:Pro Pro:Fru:Gly B:Gly:Treh B:GPC:Sor:Tau:U	1 h	Direct freezing	7 days
2. Effect of freezing method	Gly Gly:Treh	1 h	Direct freezing Controlled-rate freezing	7 days
3. Effect of pre-incubation duration	Gly Gly:Treh	30 min, 1 h, 3 h	Controlled-rate freezing	7 days
4. Effect of storage duration	Gly Gly:Treh	30 min	Controlled-rate freezing	7 days 32 days

**Table 3:** Post-thaw survival of *S. parasitica* after seven days of freezing in different 10% (*v*/*v*) DESs and glycerol. Survival is expressed as the number of biological replicates showing visible post-thaw mycelial regrowth out of three biological replicates (*n* = 3).

Solvent	Survival
Gly	3/3
Pro:Gly	3/3
Ect:Gly	3/3
B:Gly	3/3
Gly:Treh	3/3
B:GPC:Sor:Tau:U	3/3
B:Gly:Treh	2/3
B:Gly:Suc	2/3
B:Gly:Ect	1/3
B:Suc:Pro	1/3
Sar:Gly	0/3
DMSP:Gly	0/3

## Data Availability

The raw measurements of mycelial radius used for statistical analysis are provided in Supplementary Dataset S1. The statistical output, including Kruskal–Wallis tests, group summaries, and Dunn’s post hoc tests with Holm correction, is provided in Supplementary Dataset S2.

## Supplementary Materials

The following supporting information can be downloaded at: https://www.mdpi.com/article/10.3390/mps9030085/s1, Dataset S1: Raw measurements of mycelial radius in cm used for statistical analysis. Dataset S2: Statistical output including Kruskal–Wallis tests, group summaries, and Dunn’s post hoc tests with Holm correction.
